# Differential Responses of Plasma Adropin Concentrations To Dietary Glucose or Fructose Consumption In Humans

**DOI:** 10.1038/srep14691

**Published:** 2015-10-05

**Authors:** Andrew A. Butler, Marie-Pierre St-Onge, Emily A. Siebert, Valentina Medici, Kimber L. Stanhope, Peter J. Havel

**Affiliations:** 1 Department of Pharmacology and Physiology, Saint Louis University School of Medicine, 1402 South Grand Boulevard, St Louis, Missouri 63104, USA; 2 College of Physicians and Surgeons, Columbia University, New York, NY, USA; 3 Division of Gastroenterology, School of Medicine , University of California at Davis, 1 Shields Ave, Davis, CA 95616; 4 Departments of Nutrition and Molecular Biosciences, School of Veterinary Medicine, University of California at Davis, 1 Shields Ave, Davis, CA 95616.

## Abstract

Adropin is a peptide hormone encoded by the *Energy Homeostasis Associated* (*ENHO*) gene whose physiological role in humans remains incompletely defined. Here we investigated the impact of dietary interventions that affect systemic glucose and lipid metabolism on plasma adropin concentrations in humans. Consumption of glucose or fructose as 25% of daily energy requirements (E) differentially affected plasma adropin concentrations (P < 0.005) irrespective of duration, sex or age. Glucose consumption reduced plasma adropin from 3.55 ± 0.26 to 3.28 ± 0.23 ng/ml (N = 42). Fructose consumption increased plasma adropin from 3.63 ± 0.29 to 3.93 ± 0.34 ng/ml (N = 45). Consumption of high fructose corn syrup (HFCS) as 25% E had no effect (3.43 ± 0.32 versus 3.39 **± **0.24 ng/ml, N = 26). Overall, the effect of glucose, HFCS and fructose on circulating adropin concentrations were similar to those observed on postprandial plasma triglyceride concentrations. Furthermore, increases in plasma adropin levels with fructose intake were most robust in individuals exhibiting hypertriglyceridemia. Individuals with low plasma adropin concentrations also exhibited rapid increases in plasma levels following consumption of breakfasts supplemented with lipids. These are the first results linking plasma adropin levels with dietary sugar intake in humans, with the impact of fructose consumption linked to systemic triglyceride metabolism. In addition, dietary fat intake may also increase circulating adropin concentrations.

Rapid effects of fasting and feeding on adropin expression in mice suggest functions related to metabolic adaptation. Consistent with this hypothesis, adropin-knockout mice are insulin resistant^1^. Adropin therapy enhances glucose clearance, improves hyperinsulinemia and enhances muscle insulin action in diet-induced obese (DIO) mice^2^,^3^. Adropin may defend against body obesity, with adropin therapy and overexpression producing lean phenotypes^2^. Adropin also functions as a nutrient-sensing peptide hormone signaling nutrient/caloric sufficiency to promote glucose utilization and limiting fat oxidation in skeletal musce^4^. It is important to note other biologic functions that may also potentially affect health including endothelial function^5^ and development of the nervous system^2^,^6^.

The roles of adropin in human physiology are not as clear. In women, we recently reported a positive association between plasma adropin concentrations and dietary fat consumption^7^ and increased plasma adropin concentrations at 3 and 6 months following Roux-en-Y gastric bypass^8^. Fish oil supplements added to a high sugar diet increased plasma adropin concentrations in nonhuman primates^9^. These intriguing results suggest a link between diet and circulating adropin levels. However, whether diet affects plasma adropin concentrations in humans is unclear.

To address this gap in knowledge, plasma adropin concentrations were measured in samples obtained at baseline and after consumption of glucose, fructose or high fructose corn syrup (HFCS) as 25% of energy (E) for 2 wk (Study A)^10^, or glucose or fructose as 25%E for 10 wk (Study B)^11^,^12^. These studies were designed to determine whether fructose consumption affects cardiovascular and metabolic risk factors. As dietary fat alters adropin expression in mice^1^,^2^,^13^,^14^, we investigated whether dietary fat intake would increase plasma adropin concentrations in humans using samples collected after consumption of a breakfast meal containing medium- or long-chain triglycerides (TG) (Study C)^15^.

## Results

Biometrics for the participants of Studies A and B whose plasma were used in the current experiments are shown in Table 1. Study A subjects consuming glucose, fructose or HFCS gained 0.4 kg (*P *< 0.05, paired t test), 0.1 kg, and 0.8 kg (*P *< 0.05, paired t test) body weight, respectively (Table 1)^10^. In Study B, fructose and glucose consumption had comparable effects on body weight (Table 1), but had different effects on regional body fat distribution^11^. Glucose consumption increased subcutaneous adipose tissue volume, while fructose consumption resulted in a preferential expansion of visceral adipose tissue as assessed by computerized tomography.

**Figure 1:**
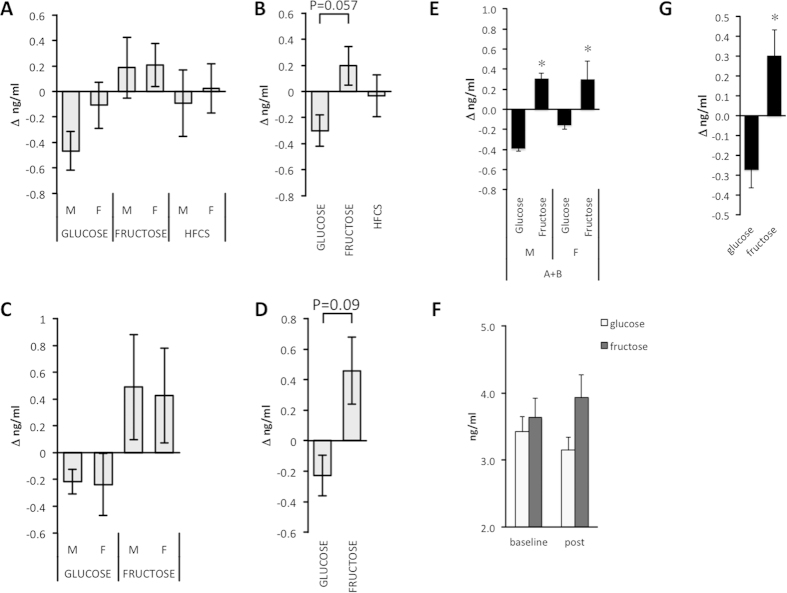
Effects of sugar consumption on plasma adropin concentrations. (**A**) The change (Δ) in plasma adropin concentrations is shown for males (M, N = 43) and females (F,N = 39) who consumed glucose (N = 28; 15M, 13F), fructose (N = 28; 15M, 13F) or HFCS (N = 26, 13M, 13F) for 2 wk (Study A). **(B)** There was no significant effect of sex; data grouped by sugar type only are also shown. **(C)** Change (Δ) in plasma adropin concentrations is shown for males (M, N = 15) and females (F, N = 16) who consumed glucose (N = 14; 6 M, 8 F) or fructose (N = 16; 9M, 8F) for 10 wk (Study B). **(D)** There was no significant effect of sex; data from Study B grouped are also shown grouped by sugar type. **(E–F)** Data pooled from Study A and B for the glucose and fructose groups. **(E)** Males (M, N = 45) and females (F, N = 42) exhibited similar responses to glucose (N = 42) or fructose (45) consumption; *P < 0.01. **(F)** Plasma adropin levels at baseline were similar for the glucose and fructose groups, but then diverged with the consumption of glucose or fructose as 25% of daily energy requirements. **(G)** The difference in the effect of glucose or fructose consumption on plasma adropin levels was highly significant. *P < 0.01.

Plasma adropin concentrations at baseline and after dietary intervention are shown for Study A and Study B (Table 2). Baseline adropin concentrations were similar between groups in both studies. For Study A, there was a trend for significant interaction between sugar and time (F_2,75 _= 2.830, P = 0.069), although the effect of time was not significant (F_2,75 _= 0.898, P > 0.05). Analysis of the change of plasma adropin concentrations (Δ^Adropin^) indicated that glucose and fructose consumption were having opposite effects (Fig. 1A,B), with a trend (F_2,75 _= 1.683, P =0.061) for a difference between the three groups. Post hoc analysis indicated a tendency (P = 0.057) for a difference, with fructose consumption increasing and glucose consumption reducing plasma adropin concentrations (Fig. 1B). Plasma adropin concentrations were not significantly affected by HFCS (Fig. 1A,B). While glucose appeared to have a more pronounced effect on plasma adropin concentration in males (Fig. 1A), there was no significant effect of sex.

A similar differential response to glucose and fructose was observed in Study B. Again, there was a strong trend for an interaction between diet and sugar (F_1,26 _= 3.320, P = 0.08) with no effect of time (F_1,26 _= 1.741, P > 0.05). As before, there was a trend for a difference in Δ^Adropin^ between the glucose (reduced) and fructose (increased) groups (F1, 26 = 3.055, P = 0.092) that was not affected of by sex (Fig. 1C,D).

As the effects of glucose and fructose consumption on plasma adropin concentrations were similar in the two studies, we performed an additional analysis using combined glucose and fructose data with study, age, BMI and ethnicity as covariates. The interaction between sugar and time was now highly significant (F_1,76 _= 10.914, P = 0.001), with no significant effect of time (F_1,76 _= 0.906, P > 0.05) (Fig. 1E,F). The other parameters had no impact on the effects of sugar (effect of age, F_1,76 _= 0.020, P > 0.05; BMI, F_1,76_=1.202, P>0.05; ethnicity, F_1,76 _= 0.054, P > 0.05; sex, F_1,76 _= 0.047, P > 0.05; and study, F_1,76 _= 0.072, P > 0.05). A univariate analysis comparing Δ^Adropin^ between glucose and fructose with study, sex, age, BMI and ethnicity as covariates also indicated a highly significant difference (F_1,75 _= 9.994, P < 0.005) (Δ^Adropin^ in ng/ml for glucose, −0.26 ± 0.15 ng/ml, 95% CI −0.55, +0.04, N = 42; fructose, +0.32 ± 0.13 ng/ml, 95% CI −0.06, +0.58, N = 45) (Fig. 1G).

### A positive interaction between baseline TG levels and fructose consumption on plasma adropin concentrations

**Figure 2:**
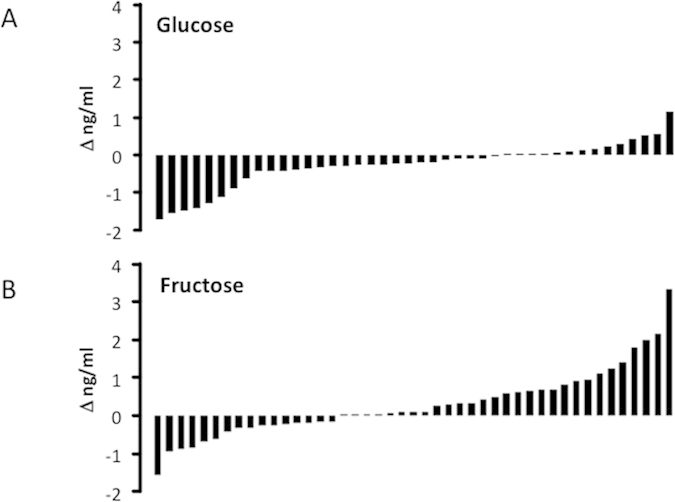
Individuals vary in their response to glucose or fructose consumption. (**A**) Change in plasma adropin concentrations (Δ ng/ml) with glucose consumption in individuals. (**B**) Change in plasma adropin concentrations with fructose consumption in individuals. The data shown in panels (**A,B**) are combined from Study (**A**) and Study (**B**).

Inspection of Δ^Adropin^ for Study A and B participants consuming glucose or fructose indicated a responder/non-responder scenario (Fig. 2). With glucose consumption, approximately 2/3 of the group (27/42) exhibited a decline in plasma adropin (Fig. 2A). With fructose consumption, plasma adropin levels increased in ~60% of the subjects (25/45) (Fig. 2B).

A large body of data related to lipid and glucose metabolism had been collected from all participants during the original experiments^10^,^11^. We therefore examined whether responders and nonresponders are distinguished by differences in risk factors for cardiovascular disease and type 2 diabetes. This initially involved an unbiased screen for associations between the plasma adropin concentrations (baseline, post-intervention and Δ^Adropin^) and previously collected plasma lipid and glucose metabolic parameters. To differentiate between responders and non-responders, we separated data pooled from Study A and B into quartiles ranked by Δ^Adropin^. To determine whether the differences in Δ^Adropin^ in each quartile correlated with plasma lipid and lipoprotein risk factors, we performed an initial screen of the risk factors comparing baseline values in the 1^st^ and 4^th^ quartiles using a Student’s t-test. Factors that showed significant differences (P < 0.05) in Δ^Adropin^ between the 1^st^ and 4^th^ quartile were then analyzed further using ANOVA.

**Figure 3:**
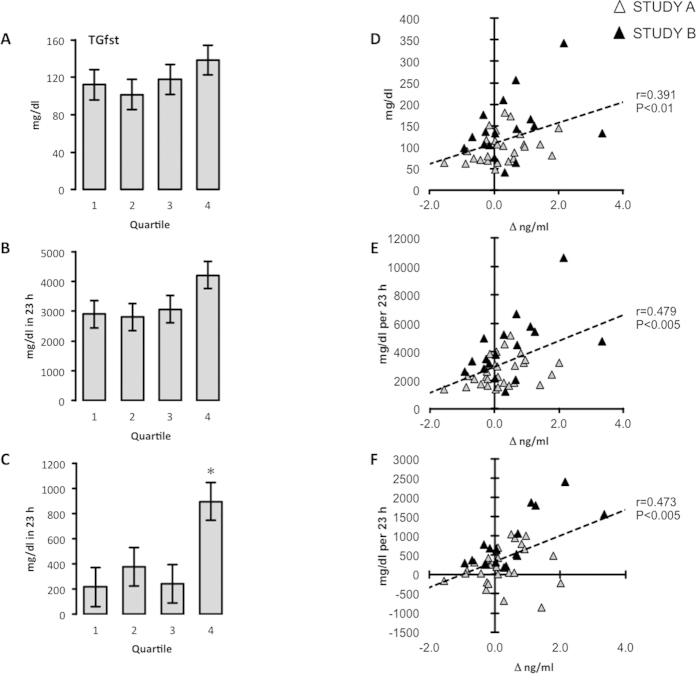
Association between the increase in plasma adropin levels with fructose consumption and fasting TG (A,D), total area under the curve for TG over 23 h (B,E) and integrative area under the curve for TG over 23 h (C,F). The TG shown in (**A**–**C**) are averages based on quartile for Δ^adropin^ adjusted for age, sex, BMI and % body fat. The quartiles are for Δ^adropin^ ranked from 1^st^ (lowest) to 4^th^ (highest). *P < 0.05 vs. 1^st^ and 3^rd^ quartile. Scatterplots shown in (**D**–**F**) have baseline TG data (fasting, total or integrative area under the curve) in the y-axis, and Δ^adropin^ in the x-axis.

This strategy did not identify distinguishing characteristics in the glucose group. However, the results of this analysis suggest differences in plasma TG between the 1^st^ and 4^th^ quartiles in participants consuming fructose. Further analysis of fasting TG (TG_fst_) (Fig. 3A), the total area under the curve (TG_totAUC_) (Fig. 3B), and the incremental area under the curve (TG_iAUC_) (Fig. 3C) indicated a higher circulating TG profile in the 4^th^ quartile group compared with the other 3 quartiles. This was significant for TG_iAUC_, which was markedly (>2-fold) higher in individuals in the 4^th^ relative to all other quartiles (P < 0.05). Distribution by age and sex was similar in the 4 groups (mean ± SE of the age for the 1^st^ to 4^th^ quartiles, 38.2 ± 5.1 yr, 32.7 ± 3.5 yr, 38.4 ± 4.2 yr and 37.8 ± 4.7 y; number of males and females in the1^st^ to 4^th^ quartiles, 7 male/4 female, 7/4, 3/8 and 6/5). There were also significant correlations between Δ^Adropin^ and baseline values for TG_Fst_ (r = 0.391, P < 0.01), TG_iAUC_ (r = 0.479, P = <0.005) and TG_AUC_(r = 0.473, P < 0.005) (Fig. 3D–F).

Of note, the most pronounced fold-increase (3.8 fold) of adropin with fructose consumption was observed in a female in Study B (age 53 yr, BMI 34.4 kg/m^2^) who also had the lowest plasma adropin concentrations measured in these studies (0.6 ng/ml) and high risk factors for cardiovascular disease. The individual had the highest TG levels at baseline (TG_fst_ 342 mg/dl, +2.9 SD from the mean for all participants in Study B; TG_iAUC_ 2411 mg/dl/23h, +2.8 SD; TG_totAUC_ 10619 md/dl/23 h, +2.9 SD). This participant also had very high levels of fasting TG- and cholesterol-rich remnant lipoproteins (RLPTG_fst_ 153.0 mg/dl, +4.4 SD from the mean of 27.7 mg/dl in Study B; RLPC_fst_ 26.6 mg/dl, +4.3 SD from the mean of 7.4 mg/dl in Study B).

### An acute effect of fat consumption on plasma adropin concentrations

**Figure 4:**
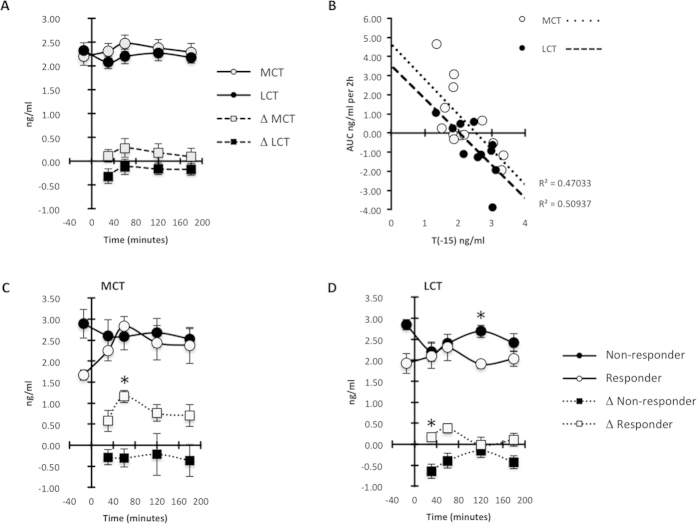
Effects of MCT or LCT consumption on plasma adropin concentrations suggests a responder/nonresponder situation. (**A**) Averages in the absolute values and delta (Δ, values at the various time points minus baseline) following MCT or LCT consumption. (**B**) Inverse association between the area under the curve (AUC) for the change in plasma adropin concentrations and baseline values taken 15 minutes before consumption of meals containing MCT or LCT. (**C**) Individuals who are “responders” exhibit a marked increase in plasma adropin concentrations after consumption of MCT when compared to “low responders”. “High responders” are characterized by having low plasma adropin concentrations at baseline, with MCT consumption increasing plasma adropin levels to that observed in “low responders”. *P < 0.05. (**D**) The distinction between responders and nonresponders following LCT consumption is less clear. While the Δ in plasma adropin was higher in responders, this was due to nonresponders showing a marked decline in adropin following consumption of the meal.

In the next experiment we examined whether dietary lipid intake would rapidly affect plasma adropin concentrations. Physical characteristics of the Study C participants have been reported previously^15^. Participants were provided a breakfast supplemented with 20 g of test oils comprised of either medium-chain or long-chain TG (MCT, LCT), in a 2-arm, randomized, single-blind, cross-over design as previously described^15^. The initial analysis of plasma adropin concentrations following consumption of MCT or LCT suggested no effect (Fig. 4A). However, further analysis of the increase in plasma adropin concentrations following the meal suggested a potential responder/nonresponder situation following MCT and LCT consumption, with an inverse association between the AUC and baseline plasma adropin concentrations (Fig. 4B). The postprandial area under the curve (AUC) for adropin also tended to be higher following MCT compared with LCT (AUC in ng/ml/2 h for MCT, 0.6 ± 0.5, for LCT, −0.5 ± 0.3; F_1,9 _= 4.081, P = 0.074). The potential responder/nonresponder situation was further analyzed by grouping the highest and lowest “responders” (AUC in ng/ml per 2 h for high responders to MCT, 2.9 ± 0.7, low responders, −1.0 ± 0.4, P < 0.01; for higher responders to LCT, 0.6 ± 0.2; low responders, −1.4 ± 0.2, P < 0.01). Interestingly, participants exhibiting the largest increase in response to MCT (“responders) also appeared to have lower plasma adropin concentrations at baseline (Fig. 4C). For LCT, when divided into low and high responders a similar difference in the AUC was evident (adropin concentrations at T_-15_ for high responders, 1.9 ± 0.2 ng/ml; for low responders, 2.8 ± 0.1 ng/ml, P < 0.05). However, the distinction between responders and nonresponders was less clear for LCT, with nonresponders showing a dip following breakfast (Fig. 4D). The differences in the response to MCT and LCT, and the ambiguity of the responder/nonresponder situation following LCT consumption, may be due to plasma samples from the two strongest responders to MCT not being available for the LCT arm of the study.

## Discussion

To our knowledge there has been only one report suggesting that plasma levels of adropin in humans are responsive to changes of energy balance, increasing after Roux-en Y gastric bypass^8^. Our data are therefore significant for being the first to suggest that diet coupled with systemic TG metabolism has an important influence in regulating plasma adropin concentrations in humans. We found that glucose consumption lowers while fructose increases plasma adropin concentrations. The differential effect of fructose and glucose was corroborated by an intermediate essentially zero effect, in Study A subjects consuming HFCS which is composed of 55% fructose and 45% glucose.

While the averaged response appears small (5–10%), there was considerable variability between individuals suggesting interactions between sugar and other variables. In the fructose group, the participants exhibiting the largest increases of plasma adropin had higher baseline circulating TG profiles. This outcome suggests an association between elevated TG levels and the regulation of plasma adropin concentrations in humans.

The consistency in the outcomes observed in the two different 2 wk and 10 wk treatment paradigms is a strength of this study. However, data interpretation is complicated by the lack of specific information regarding the molecular mechanisms by which circulating adropin levels are regulated. The observed changes are presumably indirect downstream consequences of changes in lipid and glucose metabolism associated with sugar consumption. Glucose passes through the liver and is readily oxidized as needed or converted to glycogen. In contrast, fructose is retained by the liver and is more readily converted to fatty acids and exported as very-low density lipoproteins. Consumption of diets with high fructose content has been suggested to promote risk factors for cardiovascular and metabolic risk disease. Indeed, in the studies from which these plasma samples were obtained, fructose consumption was associated with the development of dyslipidemia^10^,^11^ and insulin resistance^11^.

It is of interest to note the parallels between the effects of adropin and fructose consumption on fuel selection. In mice, adropin limits fat oxidation while enhancing glucose oxidation in lean and diet-induced obese mice^3^,^4^. In the 10wk study, fructose consumption lowered insulin sensitivity, increased *de novo* lipogenesis, reduced postprandial fat oxidation, and enhanced glucose oxidation, and, even though weight gain was similar for both groups, only fructose consumption increased intra-abdominal obesity^11^,^12^. If adropin has similar functions in humans, then the increase in plasma adropin concentrations with fructose consumption is consistent with the outcomes observed in fuel selection (i.e., reduced net postprandial fat oxidation, enhanced net postprandial carbohydrate oxidation)^11^,^12^. The positive association between high TG at baseline and the impact of fructose consumption on plasma adropin concentrations is also suggestive (Fig. 4). If adropin limits fat oxidation in humans as it does in mice, then inhibition of fat oxidation might be one of several factors contributing to elevated TG levels following fructose consumption.

Previous findings on the metabolic response of humans to MCT are, however, not consistent with this hypothesis. While MCT appear to have a stimulatory effect on plasma adropin concentrations, they are preferentially oxidized and cause a net increase in energy expenditure and fat oxidation^16^,^17^. Moreover, although plasma TG increased after consumption of MCT and LCT, the response was lower following MCT^15^. The association between fructose consumption, system TG metabolism and plasma adropin concentrations may therefore involve other indirect time-dependent mechanisms.

Whether fructose consumption would similarly affect plasma adropin concentrations in rodents as observed in humans has not been determined. It is worth noting that the provision of fructose to mice has also been observed to increase adiposity by enhancing lipogenesis and reducing energy expenditure^18^,^19^. An increase of plasma adropin would serve to facilitate the expansion of the fat reserves by reducing fatty acid oxidation while promoting the oxidation of carbohydrates. Future studies investigating whether fructose consumption affects adropin expression in mice, and whether adropin regulates fuel selection in humans, are required.

One caveat to this model is that over expression of adropin or administration of adropin peptide to mice does not result in obesity^2^. Indeed, transgenic mice over expressing adropin under the control of the human β-actin promoter exhibit delayed DIO, while treatment of DIO male B6 mice with synthetic adropin is associated with mild weight loss. In both cases, a shift away from fat oxidation towards glucose oxidation has been observed^3^,^4^. One possible explanation for this discrepancy is that the mouse models represent a non-physiological condition. In the studies with administration of synthetic adropin peptide, the doses used are pharmacological, likely resulting in spikes in plasma adropin concentrations well above what is normally observed in the fed condition. For the transgenic mice, the use of the β-actin promoter results in high levels of expression in tissues where the endogenous *Enho* gene is either not active or expressed at low levels. A mild increase in plasma adropin observed in physiological conditions may therefore be sufficient to alter fuel selection without producing changes in metabolism that prevent weight gain.

Finally, another perhaps simpler interpretation of this data is that there is a link between hypertriglyceridemia and plasma adropin levels. This might involve a homeostatic interaction between adropin and TG metabolism. In mice, adropin appears to be stimulated by dietary fat content. Adropin also has an inhibitory effect on the expression of genes involved in lipogenesis, although whether this effect is direct or secondary to other metabolic changes has not been determined^2^. The association between baseline TG levels and the increase in plasma adropin concentrations might therefore indicate an attempt to maintain homeostasis.

The second part of this study examined plasma adropin concentrations following consumption of a high fat meal. The samples used for this experiment came from a study comparing the response of adult men to a breakfast meal supplemented with 20 g of MCT or LCT^15^. The original goal of the study was to compare the satiating effects of MCT with LCT, including an analysis of gut hormone responses.

The results of our analysis suggest a complex situation with regard to the response of plasma adropin to dietary fats in humans. The impact of dietary fat on plasma adropin concentrations appears to be dependent on baseline levels, with an inverse relationship. Strong linear associations between the postprandial AUC and baseline fasting plasma adropin concentrations were evident. While this was evident for both LCT and MCT, the latter appear to have elicited a more potent effect in those individuals who are “responders”. Indeed, one interpretation of our data is that individuals with plasma adropin concentrations at the low end of the spectrum will experience a rapid increase following ingestion of meals rich in fats.

The main outcomes of this report should be considered preliminary as none of the studies from which samples were obtained were specifically designed to investigate the regulation of circulating adropin. However, these results are important in suggesting that further studies investigating the impact of macronutrient consumption on plasma adropin concentrations are needed.

These results suggest an association between dietary and systemic lipid/triglyceride metabolism in the regulation of plasma adropin concentrations. Lipids originating either from the diet or from endogenous production appear to positively affect plasma adropin concentrations in humans. Further studies designed to specifically investigate the mechanisms linking lipid metabolism to plasma adropin concentrations are warranted.

## Methods

The protocols used for original studies were reviewed and approved by the Institutional Review Boards at UC Davis^10^,^11^ and St. Luke’s/Roosevelt Hospital^15^. The studies were subsequently performed in accordance following the guidelines provided by the IRB’s at UC Davis and St. Luke’s/Roosevelt Hospital. All of the participants provided written informed consent for the use of plasma collected in the experiments. Plasma adropin concentrations at baseline from Study A and Study B were reported previously^8^.

### Study A (UC Davis CCRC, 2 wk dietary intervention examining the effect of glucose, fructose or HFCS on plasma adropin concentrations. Clinical Trials.Gov Identifier NCT01103921)

Plasma for this experiment was from 82 participants (43 males, 39 females) of mixed ethnicity (44 Caucasian, 9 Hispanic, 13 Asian, 11 African American, 3 Pacific Islander and 2 of mixed heritage). Biometric data are shown in Table 1. Recruiting, exclusion criteria and study design are published^10^. The study consisted of three phases. In phase one, study participants resided at the CCRC for a 3.5-d inpatient baseline period. During the second and third day, study participants were provided energy-balanced meals consisting of conventional foods. The diet during this period contained 55%E as complex carbohydrate, 30% fat and 15% protein served as 3 meals, with 25% of the energy provided as breakfast at 0900 h, 35% as lunch (1300 h) and 40% as dinner (1800 h). In phase two, a 12-d outpatient period, participants were provided with sugar-sweetened beverages providing 25%E split between three meals (breakfast, lunch and dinner). Participants were asked to consume one beverage per meal, to consume their normal diet, and to not to consume other sweetened beverages (including fruit juice). Phase three involved another 3.5-d inpatient period at the CCRC with participant provided energy balanced meals derived from conventional foods and sugar-sweetened beverages. Pooled samples collected at 0800, 0830 and 0900 h after an overnight fast on the third day of the baseline period and third phases were used to measure plasma adropin at baseline and after 2 wk of dietary intervention.

### Study B (UC Davis CCRC, 10 wk dietary intervention examining the effect of glucose or fructose on plasma adropin concentrations. Clinical Trials.Gov Identifier NCT01165853)

Plasma used for this study was obtained from 32 participants (16 males, 16 females; 24 Caucasian, 5 Hispanic, 3 African American) consuming 25%E from fructose or glucose for 10 wk^11^. Biometrics are presented in table 1. This study also consisted of three phases. An inpatient baseline period lasted for 2 wk where participants consumed an energy balanced diet. The second phase involved an 8 wk outpatient period, with participants provided fructose- or glucose-sweetened beverages providing 25%E. The final phase was a 2 wk inpatient period involving continued consumption of the fructose- or glucose-sweetened beverages. The meals provided during the inpatient periods were comprised of 55%E as carbohydrate, 30%E as fat and 15%E as protein. Pooled samples collected at 0800, 0830 and 0900 h after an overnight fast on the final day of the baseline period and third phases were used to measure plasma adropin.

### Study C (NYONRC, effect of medium or long chain triglyceride consumption on plasma adropin concentrations. Clinical Trials.Gov Identifier NCT01952977)

Physical characteristics have been reported^15^. The study was comprised of 5 Caucasian, 8 African American, 2 Hispanic and 2 men of mixed heritage, aged 39.4 ± 1.8y who were overweight (mean body weight, 88.9 ± 2.3 kg; mean BMI, 28.2 ± 0.3 kg/m^2^). In brief, this study was performed in two parts (C1, C2). Ten men participated in Study C1; the protocol was then revised and another 7 men enrolled into Study C2. Each study involved a 2-arm, randomized, single-blind, cross-over design. Each arm consisted of a test day spaced 3 to 14 d apart that differed in the type of oil incorporated into the meal: MCT oil (Neobee 1053, Stepan Company, Northfield, IL) or corn oil (LCT, Mazola, ACH Food Companies, Cordova, TN). The nutrient composition of the breakfast meals was different (C1: muffin with test oil added and orange juice, total energy 2671 kJ, 105 g carbohydrate, 9.6 g protein, 20 g of fat, 20 g of test oil; C2: boost shake with test oil, total energy 2510 kJ, 71.6 g carbohydrate, 17.5 g protein, 27 g of fat, 20 g of test oil). Both diets provided the same amount of test oil. For 2 d prior to the test, participants were requested not to consume alcohol or participate in structured exercise. They were asked to record their food intake at dinner the night before the test day and consume the same meal the night before the follow-up test day. The participants fasted for at least 12 h during the night before the test day.

### Plasma adropin measurements

Adropin concentrations were measured using EDTA-plasma not previously been thawed in duplicate by EIA (Peninsula Laboratories, Bachem, San Carlos, CA)^8^. The intra-assay coefficient of variation (CV) is <10%, while the inter-assay variability fluctuates between 20 to 30%.

### Statistical analysis

Data analysis used SPSS Statistics 22. Univariate analysis with repeated measures (within-subjects factors: time at pre- and post-intervention) was used to determine the impact of dietary intervention, with sugar type as between-subjects factors in the analysis. For comparisons between sugar groups involving single measurements (for example, the change in plasma adropin concentrations calculated by subtracting baseline from the post-intervention value), ANOVA were used followed by Bonferroni corrected multiple comparisons. For analyzing the response to MCT and LCT, which was a randomized cross-over design, a pair *t-*test was used for the analysis of the AUC.

## Additional Information

**How to cite this article**: Butler, A. A. *et al.* Differential Responses of Plasma Adropin Concentrations To Dietary Glucose or Fructose Consumption In Humans. *Sci. Rep.* **5**, 14691; doi: 10.1038/srep14691 (2015).

## Figures and Tables

**Table 1:** Biometrics for Study A and B participants (mean, SE).

Study	Sugar	Sex	N	Age (yr)	Weight (kg) (pre/post)	BMI (kg/m^2^) (pre/post)	Body fat (%) (pre)	BPsys (pre)	BPdiast (pre)
**A (2 wk)**	**Glucose**	M	15	26.5 ± 1.6	78.9 ± 3.3 79.5 ± 3.4	25.6 ± 0.9 25.8 ± 0.9	23.1 ± 1.6	124 ± 2	75 ± 2
		F	13	25.4 ± 1.5	71.6 ± 3.4 72.3 ± 3.6	26.0 ± 1.0 26.2 ± 1.1	35.6 ± 1.3	112 ± 3	72 ± 2
	**Fructose**	M	15	26.5 ± 1.8	79.1 ± 3.5 79.0 ± 3.5	24.6 ± 0.9 24.6 ± 0.9	22.3 ± 2.3	122 ± 2	74 ± 2
		F	13	27.2 ± 1.6	71.8 ± 3.2 72.0 ± 3.1	26.2 ± 1.0 26.3 ± 1.0	36.6 ± 1.5	111 ± 2	69 ± 2
	**HFCS**	M	13	23.8 ± 1.8	74.1 ± 3.4 75.2 ± 3.5	23.8 ± 0.9 24.1 ± 0.9	17.8 ± 1.6	120 ± 2	73 ± 2
		F	13	29.7 ± 1.6	69.2 ± 4.3 69.7 ± 4.3	25.5 ± 1.3 25.7 ± 1.3	33.9 ± 1.7	113 ± 3	72 ± 2
**B (10 wk)**	**Glucose**	M	6	55.0 ± 3.5	86.4 ± 3.1 90.3 ± 3.3	28.1 ± 1.1 28.8 ± 1.2	28.1 ± 3.8	120 ± 2	75 ± 2
		F	8	56.0 ± 1.9	83.9 ± 4.6 85.0 ± 4.9	29.4 ± 1.4 29.8 ± 1.4	42.2 ± 3.1	123 ± 2	79 ± 2
	**Fructose**	M	9	51.8 ± 3.5	88.9 ± 2.9 90.6 ± 2.8	28.5 ± 0.7 29.0 ± 0.6	26.8 ± 6.1	120 ± 2	76 ± 1
		F	8	52.9 ± 2.4	81.8 ± 4.3 82.5 ± 4.3	30.3 ± 1.1 30.6 ± 1.1	41.2 ± 5.5	120 ± 3	77 ± 3

**Table 2:** Plasma adropin concentrations at baseline and after sugar consumption.

Study	Sex	Sugar (N)	Baseline	2 wk
**A (2 wk)**	**Males**	**Glucose (15)**	3.95 ± 0.44	3.50 ± 0.37
		**Fructose (15)**	4.13 ± 0.49	4.31 ± 0.54
		**HFCS (13)**	3.74 ± 0.56	3.64 ± 0.41
	**Females**	**Glucose (13)**	3.03 ± 0.30	2.92 ± 0.19
		**Fructose (13)**	3.81 ± 0.55	4.02 ± 0.66
		**HFCS (13)**	3.12 ± 0.31	3.15 ± 0.27
	**All**	**Glucose (28)**	3.52 ± 0.28	3.23 ± 0.22
		**Fructose (28)**	3.98 ± 0.36	4.18 ± 0.42
		**HFCS (26)**	3.43 ± 0.32	3.39 ± 0.24
**B (10 wk)**	**Sex**	**Sugar (N)**	**Baseline**	**10 wk**
	**Males**	**Glucose (6)**	3.98 ± 1.21	3.75 ± 1.20
		**Fructose (9)**	3.60 ± 0.81	4.09 ± 1.00
	**Females**	**Glucose (8)**	3.35 ± 0.44	3.10 ± 0.39
		**Fructose (8)**	2.46 ± 0.42	2.90 ± 0.48
	**All**	**Glucose (14)**	3.62 ± 0.56	3.38 ± 0.54
		**Fructose (17)**	3.06 ± 0.48	3.53 ± 0.58
